# Effectiveness comparisons of acupuncture for diabetic nephropathy proteinuria: A systematic review and meta-analysis: study protocol

**DOI:** 10.1097/MD.0000000000017819

**Published:** 2019-11-22

**Authors:** Yao-Dong Miao, Wen-Yuan Gu, Zhi-Qiang Liu, Yun-Tao Ma, Sheng Deng

**Affiliations:** a Second Affiliated Hospital of Tianjin University of TCM; b First Teaching Hospital of Tianjin University of TCM; c Dongzhimen Hospital, Dongcheng District, Hai Yun Cang on the 5th ZIP, China.

**Keywords:** acupuncture, diabetic nephropathy proteinuria, protocol, systematic review

## Abstract

**Introduction:**

Diabetic nephropathy (DN) is one of the microvascular complications of diabetes (DM). Proteinuria is the most important clinical feature of DN and an independent risk factor for the progression of DN. Therefore, reducing urinary protein is the primary goal of DN treatment. Acupuncture has long been widely used in the treatment of DN. Therefore, this paper conducted a meta-analysis of the clinical efficacy of acupuncture in the treatment of DN proteinuria, in order to comprehensively analyze the role of acupuncture in the treatment of DN.

**Methods and analysis:**

We will search for PubMed, Cochrane Library, AMED, EMbase, WorldSciNet; Nature, Science online and China Journal Full-text Database (CNKI), China Biomedical Literature CD-ROM Database (CBM), and related randomized controlled trials included in the China Resources Database. The time is limited from the construction of the library to September 2019.We will use the criteria provided by Cochrane 5.1.0 for quality assessment and risk assessment of the included studies, and use the Revman 5.3 and Stata13.0 software for meta-analysis of the effectiveness, recurrence rate, and symptom scores of DN proteinuria.

**Ethics and dissemination:**

This systematic review will evaluate the efficacy and safety of acupuncture for DN proteinuria. Because all of the data used in this systematic review and meta-analysis has been published, this review does not require ethical approval. Furthermore, all data will be analyzed anonymously during the review process Trial.

**Trial registration number:** PROSPERO CRD42019139705

## 1 Introduction

Diabetic nephropathy (DN) is one of the most common and serious chronic complications of diabetes mellitus (DM),^[1]^ and proteinuria is the most important clinical feature of DN and an independent risk factor for DN progression.^[2]^ The production of microalbuminuria can increase the all-cause mortality of DM patients.^[3]^ At present, the treatment of DN is based on lifestyle changes and eating habits, control of blood sugar, blood pressure, and correction of dyslipidemia.^[4]^ In recent years, although many studies have confirmed that drugs including vitamin D,^[5]^ immunosuppressant tacrolimus^[6,7]^ have a certain effect on reducing DN proteinuria and delaying the progression of the disease, but it is still recommended in clinical practice. Only ACEI/ARB drugs. The application of ACEI/ARB drugs has its limitations. Studies have shown that ACEI/ARB is beneficial for diabetic patients with hypertension with GFR < 60 ml/minute/1.73m2 and UACR ≥ 300 mg/g Cr, which can delay their chronic kidneys. Disease (CKD) progress. However, for patients with normal blood pressure, ACEI / ARB cannot prevent the occurrence of kidney damage caused by diabetes.

Acupuncture, 1 essential part of traditional Chinese medicine (TCM), has been widely used in clinical trials recently.^[8–11]^ The theory of acupuncture believes that the location of DN is mainly in the kidney, the deficiency of kidney and the stagnation of renal collateral are the key to the pathogenesis of DN.^[12–16]^ Yin deficiency and heat, qi stagnation and blood stasis is its main pathogenic factor. Through the application of acupuncture in the treatment of DN proteinuria's unique diagnosis and treatment system, clinical efficacy is significant. Modern research has shown that effective active ingredients in acupuncture can improve the blood supply of peripheral blood vessels and achieve therapeutic purposes.^[17,18]^ Through the action mechanism of multi-faceted and multi-target, acupuncture regulates the body function as a whole and has unique advantages in the treatment of DN proteinuria. After preliminary search and analysis of database, we found that the frequency of randomized controlled (RCT) trials of acupuncture treatment in DN proteinuria has been showing an increasing trend.^[19]^ Previous clinical trials have shown that acupuncture could ameliorate pain and improve the quality of lives in patients who suffer from DN proteinuria, and these effects are sustained.^[17,20]^ However, due to the limitation of the scale and sample size of the clinical centers, the current level of evidence-based medical evidence is still not sufficient. Therefore, we hope to evaluate the efficacy and safety of acupuncture in treating DN proteinuria by using meta-analysis, which aim to provide sufficient evidence for its clinical application.

## 2 Methods

This systematic review protocol has been registered on PROSPERO as CRD42019139705. (https://www.crd.york.ac.uk/prospero/display_record.php?ID=CRD42019139705). The protocol follows the Cochrane Handbook for Systematic Reviews of Interventions and the Preferred Reporting Items for Systematic Reviews and Meta-Analysis Protocol (PRISMA-P) statement guidelines. We will describe the changes in our full review if needed.

### 2.1 Inclusion criteria

#### 2.1.1 Types of studies

This study will include all the RCTs that relate to acupuncture therapy in treating DN proteinuria. For the included trials, the investigators need to precisely report the stochastic methods, acupuncture treatment details and parameters, diagnostic criteria, and efficacy evaluation they based on. No limitation to whether it is published or not. The experiment is limited to humans. Language is limited to Chinese and English.

#### 2.1.2 Types of participants

Participants who were definitely diagnosed with DN proteinuria would be included. The cases which relate to prostatic hyperplasia, prostate cancer or other prostate-related diseases would be excluded. In addition, there are no limitation in region, citizenship, nationality, and source of cases.

#### 2.1.3 Types of interventions

*Experimental interventions*: The intervention will include all piercing acupuncture, including hand-acupuncture, electroacupuncture, fire needle, plum blossom needle, abdominal needle and so on. Other non-piercing acupunctures such as acupressure, acupressure, acupressure, moxibustion, etc. will be excluded. Acupoint injections will also be excluded. Pharmaco-acupuncture-true and acupoint injection will be rejected, we consider that their methods and theories are different from TCM. The treatment duration and frequency are not limited.

*Control interventions*: The control interventions will include a placebo, a virtual acupuncture, a conventional drug such as an alpha blocker, an antibiotic, a botanical preparation, a non-soul anti-inflammatory drug, and the like. However, RCT of acupuncture combined with drugs or other Chinese medicine methods will be excluded.

#### 2.1.4 Types of outcome measures

*Primary outcomes*: Primary outcomes included 24-hour Urine protein quantitation, Urinary albumin excretion rate (UAER), Fasting blood glucose (FBG), Glycosylated hemoglobin (HbAlc), and Total effective rate.

*Secondary outcomes*: The second outcome measure is based on acupuncture syndrome evaluation criteria.

(1) Healing: The clinical symptoms and signs of acupuncture disappear or almost disappear, and the syndrome score is reduced by ≥90%;
(2) Significant effect: The clinical symptoms and signs of acupuncture are obviously improved, and the syndrome score is reduced by ≥60%;
(3) Effective: Chinese medicine Clinical symptoms and signs have improved, syndrome scores decreased by <60%, but ≥30%;
(4) Invalid: The clinical symptoms and signs of acupuncture were not improved, even worse, and the syndrome score was reduced by <30%. Integral variation formula (Nimodipine method: [(pre-treatment score - post-treatment score) ÷ pre-treatment score] × 100%.

### 2.2 Data source

Database Search: PubMed, Cochrane Library, AMED, EMbase, World SciNet, Nature, Science online and China National Knowledge Infrastructure (CNKI), China Biomedical Literature CD-ROM Database (CBM), China Resources Database. Search for clinical research literature on acupuncture DN proteinuria published in domestic and foreign biomedical journals from the establishment of the library to September 2019. Based on the standards of the Cochrane Collaboration Workbook of the International Evidence-Based Medicine Center, a manual and computer-based method will be used to conduct related literature searches. The search terms include: DN or Diabetic kidney disease or Diabetic proteinuria, acupuncture, acupuncture therapy, acupuncture, electroacupuncture, fire needle, plum blossom needle, skin needle, abdominal needle. Manually search for topics, abstracts, etc related to the research of Chinese Journal of Male Science, Chinese Journal of Male Science, Chinese Acupuncture and Acupuncture. The complete PubMed search strategy is summarized in Table 1.

**Table 1:**
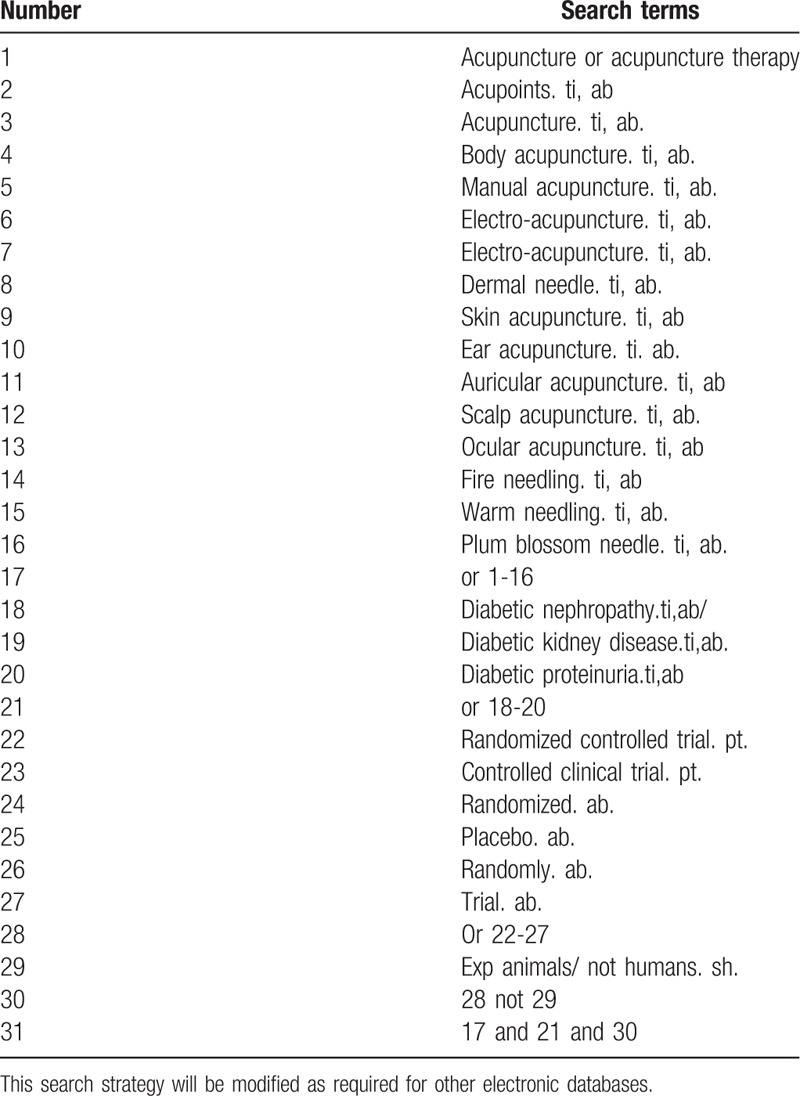
Search strategy used in PubMed database.

### 2.3 Data collection and analysis

#### 2.3.1 Selection of studies

Two investigators used EndnoteX7 software to conduct a preliminary assessment of the title and abstract of each document in the database based on the established criteria for inclusion in the study to select eligible studies. After a preliminary assessment, the full text of the selected literature would be evaluated, and the uncontrolled study, no randomization, inconsistent evaluation criteria, and similar data would be excluded. Any differences in screening that occurred during the screening study would be discussed in order to get consensus, if it still cannot be resolved, then the third author would be intervened.

#### 2.3.2 Data extraction and management

**Figure 1:**
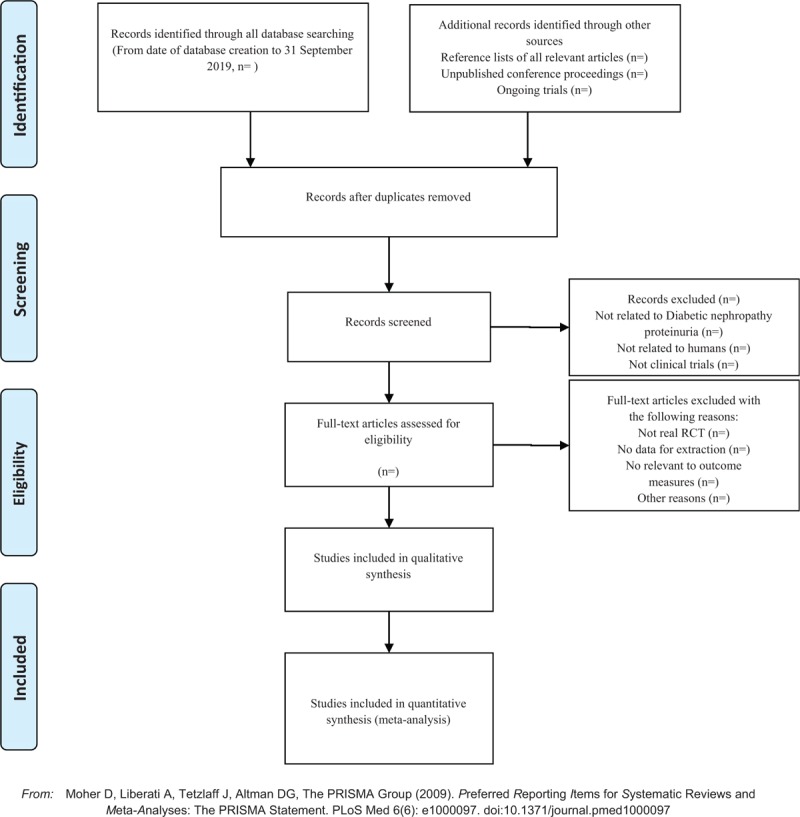
The PRISMA flow chart.

Two investigators independently extracted information from the included literature. The extracted content includes research design, random hiding and blinding, basic information of the included cases, intervention methods, observation indicators and test results of the treatment group and the control group. The extracted literature data will be filled in a unified data statistics table. For studies that provide baseline and post-treatment data, we will estimate the change values by the method recommended by Cochrane. The details of selection process will be shown in the PRISMA flow chart (Fig. 1).

#### 2.3.3 Assessment of risk of bias in included studies

Two investigators will independently evaluate the methodological quality of the included literature by using the Cobrane Collaboration's ROB tool which includes whether the random method is correct, whether blinding is used, whether it is hidden, whether it is lost or quit, whether it uses Intent-To-Treat (ITT) analysis, whether the data results are accurate, and other risks of bias. According to the relevant standards in the Cochrane Intervention System Evaluation Manual, it will be divided into low risk, high risk and unclear.

#### 2.3.4 Dealing with missing data

In the event of data loss during the screening and extraction of literature data, first, we will actively investigate the cause of data loss. Then we will contact the experimental research author by telephone, mail, etc to achieve the purpose of supplementing the missing data. If the lost data cannot be retrieved, we will only extract and analyze the useful data, besides, we will indicate the situation.

#### 2.3.5 Statistical analysis

The numerical variable will be expressed as the normalized mean difference (SMD) with a confidence interval (CI) of 95%.The heterogeneity of each pairwise comparison will be tested by chi-square test (test level α = 0.1).If there is no heterogeneity, a fixed effect model will be used. If there is significant heterogeneity between a group of studies, we will explore the reasons for the existence of heterogeneity from various aspects such as the characteristics of the subjects and the degree of variation of the interventions. Sensitivity analysis or meta-regression and subgroup analysis to explore possible sources of heterogeneity when necessary. We will use qualitative analysis of the funnel plot and graph symmetry to assess publication bias. Quantitative methods such as Begg testing and Egger testing will be used to help assess publication bias in the application.

#### 2.3.6 Assessment of heterogeneity

If there is significant heterogeneity between a group of studies, we will explore the reasons for the existence of heterogeneity from various aspects such as the characteristics of the subjects and the degree of variation of the interventions. Sensitivity analysis or subgroup analysis is performed as necessary to explain heterogeneity.

#### 2.3.7 Assessment of publication bias

The forest map and funnel plot were drawn and analyzed using Rev Man5.3 software, and the funnel plot was used to analyze potential publication bias.

#### 2.3.8 Grading the quality of evidence

The quality of evidence for the main outcomes will also be assessed with the GRADE approach. The evaluation included bias risk; heterogeneity; indirectness; imprecision; publication bias. And each level of evidence will be made “very low,” “low,” “moderate,” or “high” judgment.

## 3 Discussion

At present, there are vast of therapies in treating DN proteinuria, however, the efficacy is still unsatisfactory due to the particularity of the anatomical structure of kidney.^[21]^ Studies have shown that drug intervention protect the renal interstitial and glomeruli to a certain extent, but there is no single drug can continue to significantly ameliorate all symptoms of DN proteinuria patients.^[22–23]^ acupuncture has a profound theoretical foundation and abundant clinical experience in the treatment of DN proteinuria. Acupuncture therapy mainly achieves therapeutic effects by stimulating the body's righteousness and regulating the balance of qi and blood. In recent years, acupuncture therapy has been widely used in clinical trials of DN proteinuria. Recent studies have shown that acupuncture can alleviate the pain caused by DN proteinuria to a certain extent and improve the quality of lives of patients.^[24]^

Although abundant studies have evaluated the effectiveness of acupuncture in treating DN proteinuria, evaluation, and comparison between various treatments are still insufficient. To the best of our knowledge, a systematic review and meta-analysis has not been used in recent years to compare the effectiveness of acupuncture in the treatment of DN proteinuria. The results of meta-analysis can provide a possible ranking for acupuncture treatment of DN proteinuria. We hope that the results will provide clinicians with the best options for treating DN proteinuria and provide them with research directions. Due to the limited number of relevant high-quality studies and the few sample sizes included, the strength of the arguments of the conclusions is to some degree limited. Therefore, we hope that more large-scale, high-quality RCT should be necessary in the future. Besides, improving the quality of the original research and conducting high-quality multi-center RCT to explore the clinical efficacy of acupuncture treatment of CP/CPPS is also indispensable, through which could make the conclusion more objective and reasonable.

## Author contributions

**Data curation:** Wen-Yuan Gu.

**Formal analysis:** Wen-Yuan Gu.

**Project administration:** Zhi-Qiang Liu, Yun-Tao Ma.

**Resources:** Yun-Tao Ma.

**Software:** Zhi-Qiang Liu.

**Supervision:** Yun-Tao Ma.

**Writing – original draft:** Sheng Deng.

**Writing – review & editing:** Sheng Deng.
